# Large-Scale Molecular Epidemiological Survey of *Blastocystis* sp. among Herbivores in Egypt and Assessment of Potential Zoonotic Risk

**DOI:** 10.3390/microorganisms12071286

**Published:** 2024-06-25

**Authors:** Doaa Naguib, Nausicaa Gantois, Jeremy Desramaut, Ruben Garcia Dominguez, Nagah Arafat, Samar Magdy Atwa, Gaël Even, Damien Paul Devos, Gabriela Certad, Magali Chabé, Eric Viscogliosi

**Affiliations:** 1 CNRS, Inserm, CHU Lille, Institut Pasteur de Lille, U1019—UMR 9017—CIIL—Centre d’Infection et d’Immunité de Lille, University of Lille, F-59000 Lille, France; doaanaguib246@yahoo.com (D.N.); nausicaa.gantois@pasteur-lille.fr (N.G.); jeremy.desramaut@pasteur-lille.fr (J.D.); damienpdevos@gmail.com (D.P.D.); gabriela.certad@pasteur-lille.fr (G.C.); magali.chabe@univ-lille.fr (M.C.); 2 Department of Hygiene and Zoonoses, Faculty of Veterinary Medicine, Mansoura University, Mansoura 35516, Egypt; 3 Centro Andaluz de Biología del Desarrollo, CSIC, Universidad Pablo de Olavide, 41013 Sevilla, Spain; ruben.garcia.d@hotmail.com; 4 Department of Poultry Diseases, Faculty of Veterinary Medicine, Mansoura University, Mansoura 35516, Egypt; nagaharafat@yahoo.com; 5 Department of Internal Medicine and Infectious Diseases, Faculty of Veterinary Medicine, Mansoura University, Mansoura 35516, Egypt; samaratwa84@yahoo.com; 6 Department of Veterinary Clinical Sciences, Faculty of Veterinary Medicine, Jordan University of Science and Technology, Irbid P.O. Box 3030, Jordan; 7 GD Biotech—Gènes Diffusion, F-59000 Lille, France; g.even@genesdiffusion.com; 8 PEGASE-Biosciences (Plateforme d’Expertises Génomiques Appliquées aux Sciences Expérimentales), Institut Pasteur de Lille, F-59000 Lille, France; 9 Délégation à la Recherche Clinique et à l’Innovation, Groupement des Hôpitaux de l’Institut Catholique de Lille, F-59000 Lille, France

**Keywords:** *Blastocystis* sp., intestinal protozoa, herbivores, Africa, Egypt, molecular epidemiology, transmission, zoonosis

## Abstract

Given the proven zoonotic potential of the intestinal protozoan *Blastocystis* sp., a fast-growing number of surveys are being conducted to identify potential animal reservoirs for transmission of the parasite. Nevertheless, few epidemiological studies have been conducted on farmed animals in Egypt. Therefore, a total of 1089 fecal samples were collected from herbivores (sheep, goats, camels, horses, and rabbits) in six Egyptian governorates (Dakahlia, Gharbia, Kafr El Sheikh, Giza, Aswan, and Sharqia). Samples were screened for the presence of *Blastocystis* sp. by real-time PCR followed by sequencing of positive PCR products and phylogenetic analysis for subtyping of the isolates. Overall, *Blastocystis* sp. was identified in 37.6% of the samples, with significant differences in frequency between animal groups (sheep, 65.5%; camels, 62.2%; goats, 36.0%; rabbits, 10.1%; horses, 3.3%). Mixed infections were reported in 35.7% of the *Blastocystis* sp.-positive samples. A wide range of subtypes (STs) with varying frequency were identified from single infections in ruminants including sheep (ST1–ST3, ST5, ST10, ST14, ST21, ST24, ST26, and ST40), goats (ST1, ST3, ST5, ST10, ST26, ST40, ST43, and ST44), and camels (ST3, ST10, ST21, ST24–ST26, ST30, and ST44). Most of them overlapped across these animal groups, highlighting their adaptation to ruminant hosts. In other herbivores, only three and two STs were evidenced in rabbits (ST1–ST3) and horses (ST3 and ST44), respectively. The greater occurrence and wider genetic diversity of parasite isolates among ruminants, in contrast to other herbivores, strongly suggested that dietary habits likely played a significant role in influencing both the colonization rates of *Blastocystis* sp. and ST preference. Of all the isolates subtyped herein, 66.3% were reported as potentially zoonotic, emphasizing the significant role these animal groups may play in transmitting the parasite to humans. These findings also expand our knowledge on the prevalence, genetic diversity, host specificity, and zoonotic potential of *Blastocystis* sp. in herbivores.

## 1. Introduction

*Blastocystis* sp. is a protozoan belonging to the highly diversified group of Stramenopiles and is currently identified as the most common single-celled intestinal eukaryote in humans [1]. Consequently, it is estimated that over 1 billion people worldwide are colonized by this microorganism [2]. As with other intestinal protozoa, the primary mode of transmission of *Blastocystis* sp. is the fecal–oral route, mainly through the consumption of contaminated water and eventually food [3,4]. As a result, its prevalence recorded in developing countries, especially in Africa, may well exceed 50% in the human cohorts analyzed due to the poor sanitary conditions encountered and the unavailability of effective water treatment, facilitating its transmission [5,6,7,8]. Strikingly, *Blastocystis* sp. has also been identified possibly with a high frequency in the digestive tracts of a very wide range of domestic and captivated and free-ranging wild animals around the world, from mammals and birds to fish and insects, among others, as recently described [9,10,11,12,13,14,15].

The high prevalence of *Blastocystis* sp. in the human population obviously raised questions about its potential pathogenicity and thus its clinical relevance and risk to public health. Although the majority of individuals colonized by this protozoan are asymptomatic, several clinical cases detailed in the literature have highlighted an association between *Blastocystis* sp. infection and the development of gastrointestinal disorders [16,17] such as diarrhea and intestinal pain, as well as dermatological symptoms such as acute or chronic urticaria [18]. In vitro experiments and in vivo studies conducted in murine models have also clearly demonstrated the deleterious impact of certain *Blastocystis* sp. isolates on the host intestinal epithelium through the disruption of the epithelial barrier, together with the identification of virulence factors including cysteine proteases [19,20]. Moreover, the influence of *Blastocystis* sp. on the gut microbiota composition of the human or animal host was shown to be even beneficial through an association with increased bacterial richness or conversely deleterious by a decrease in beneficial bacteria depending on the genetic diversity of the parasite isolates [21,22,23,24,25,26]. Recent publications also highlight a direct link between *Blastocystis* sp. infection and alterations of human cognitive function likely through the ability of the parasite to produce tryptophan leading to effects on the brain [27,28].

A defining feature of the *Blastocystis* genus lies in its considerable genetic diversity revealed by the comparative analysis of small subunit (SSU) rRNA gene sequences of numerous isolates obtained from human and various animal hosts. To date, 46 lineages, so-called subtypes (STs), have been proposed [29,30,31], but 4 of them (ST18–ST20 and ST22) were considered invalid since they were the likely outcome of experimental artifacts [32]. Moreover, two STs (ST10 and ST42) were recently subdivided into two subgroups (ST10a/ST10b and ST42a/ST42b, respectively) to differentiate sequence variants within these two clusters [31]. Of those 42 legitimate STs, most of them were identified in various animal groups, and only 17 STs have been reported in humans (ST1–ST10, ST12, ST14, ST16, ST23, ST35, and ST41) with highly variable frequency [6,8,29,33,34,35,36,37]. Indeed, while infections with certain STs such as ST12, ST16, ST23, ST35, or ST41 remain sporadic, over 90% of subtyped human isolates belonged to ST1–ST4, reflecting a widespread human-to-human transmission, even though these latter four STs have also been detected in different animal groups [12]. Interestingly, several STs considered to be of animal origin and predominantly occurring in bovid (ST10 and ST14), pigs (ST5), birds (ST6 and ST7), and non-human primates (ST8) [12,13,38,39] were also found in human cohorts with a significant frequency. For instance, the avian ST6 and ST7 could represent around 10% of the isolates identified in humans outside Europe [1], whereas ST10 and ST14 have been increasingly reported in the population as part of recent surveys conducted in Asia and Africa [6,7,8,40], strongly suggesting zoonotic transmission events from different animal groups. The zoonotic potential of *Blastocystis* sp. was undoubtedly evidenced by the molecular identification of the same genetic variants belonging to either ST1, ST5, ST6, or ST8 circulating simultaneously in the same area among captive primates and their zookeepers [41,42], poultry and slaughterhouse staff members [43], or pigs and in-contact humans from commercial intensive piggeries [44]. The animal-to-human transmission of these STs was obviously related to repeated and direct contact between animals and their handlers.

Given the impact of *Blastocystis* sp. in the population and its zoonotic potential, numerous epidemiological surveys have been carried out worldwide in various animal groups with close contact with humans, in order to identify all potential reservoirs of transmission [12,13,15,39]. However, such studies are still very limited on the African continent, as for instance, in Egypt. Indeed, despite a prevalence of *Blastocystis* sp. in humans shown to exceed 70% in the north of this country by using molecular detection methods [8], only five surveys have been conducted to date in animals, mainly focusing on poultry, cattle, and pets [45,46,47,48,49]. Globally, the obtained epidemiological data reported an active circulation of the parasite in poultry and cattle, implying that both animal groups could play a role as reservoirs for zoonotic transmission. In contrast, the low frequency or absence of *Blastocystis* sp. in dogs and cats strongly suggests that pets represent poor host species for the parasite and pose a minimal zoonotic risk of infection to their owners. To complete this overview and improve our knowledge of the molecular epidemiology and circulation of the parasite, a large-scale survey was carried out in six Egyptian governorates (Sharqia, Aswan, Giza, Dakahlia, Kafr El Sheikh, and Gharbia) with the aim of determining the prevalence and ST distribution of *Blastocystis* sp. in five major groups of animals in terms of population, namely, camels, horses, rabbits, goats, and sheep, all of which live in close contact with the population and may therefore represent potential sources of zoonotic transmission.

## 2. Materials and Methods

### 2.1. Ethics Statement

All the fieldwork in this study was carried out in compliance with the Guide for the Care and Use of Laboratory Animals in Egypt and approved by the Research Ethical Committee of the Faculty of Veterinary Medicine, Mansoura University, with the code number MU-ACUC (VM.R.23.12.133). The owner’s permission was obtained before the collection of fecal specimens.

### 2.2. Animal Feces Collection

**Figure 1:**
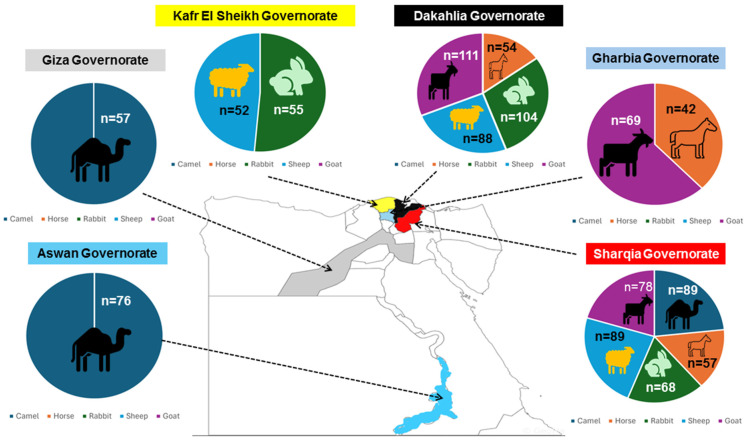
Map of Egypt displaying the number of fecal samples collected from goats, sheep, camels, horses, and rabbits in each of the six governorates surveyed.

From January to May 2023, we randomly collected a total of 1089 fresh animal fecal specimens from goats (*Capra hircus*) (*n* = 258), sheep (*Ovis aries*) (*n* = 229), rabbits (*Oryctolagus cuniculus*) (*n* = 227), horses (*Equus caballus*) (*n* = 153), and camels (*Camelus dromedarius*, often referred to as the Arabian camel) (*n* = 222) reared in about a total of thirty farms located in northern (Dakahlia, Gharbia, Kafr El Sheikh and Sharqia governorates), middle (Giza governorate), and southern (Aswan governorate) Egypt (Figure 1).

The selection of farms for sampling was based on the agreement of breeders to participate in the survey. For each animal included in the study, epidemiological data were recorded such as sex, age, location of the farm, and fecal consistency (with or without diarrhea). Goats and sheep were raised in both pastures and semi-intensive groups. Rabbits were raised in semi-intensive groups as well. Camels were kept in small groups, while horses were individually housed by farmers. Samples were collected in the winter and spring, with temperatures being similar across all governorates, except for Aswan, which is known for its hot climate. One fecal sample per animal was collected either directly from the rectum of the animal using sterile gloves or immediately after defecation onto the ground using disposable spoons. Each animal specimen was placed into a sterile plastic cup and immediately transferred to Mansoura University under complete aseptic conditions. Approximately 2 g from each fresh animal stool sample was added to 2 mL of 2.5% potassium dichromate (*w*/*v* in water) (Sigma Life Sciences, Saint-Louis, MO, USA) in a sterile tube, thoroughly mixed by vortex, and then stored at 4 °C until transfer to the Pasteur Institute of Lille, France, for further processing.

### 2.3. DNA Extraction and Molecular Detection of Blastocystis sp.

Upon arrival in France, all fecal samples stored in potassium dichromate were washed with distilled water through several centrifugation steps at 3000× *g* for 10 min. After removing the final supernatant and any traces of potassium dichromate, the remaining pellet was diluted with 1 mL of sterile water. Genomic DNA was extracted from 500 μL of the diluted pellet using the NucleoSpin 96 Soil kit (Macherey-Nagel GmbH & Co KG, Düren, Germany) following the manufacturer’s instructions. Extracted DNA was eluted in 100 μL of the elution buffer provided in the kit and stored at −20 °C until being analyzed. The presence of *Blastocystis* sp. in animal feces was assessed following the protocol of a real-time polymerase chain reaction (qPCR) assay allowing the amplification of a fragment of approximately 300 bp of the SSU rDNA gene of the parasite [50]. The designed primer pair (sense BL18SPPF1/antisense BL18SR2PP) proved to be specific to the *Blastocystis* genus and hence able to amplify the homologous domain of all parasite STs. All qPCRs using 2 μL of extracted DNA were carried out in duplicate on a Rotor-Gene Q Real-Time PCR thermocycler (Qiagen, Courtaboeuf, France). Positive (DNA from *Blastocystis* sp. ST8 axenic culture) and negative (DNA replaced by water in the PCR mixture) controls were included in each round of qPCR.

### 2.4. Sequence Analyses and Genotyping of Isolates

Positive-qPCR products were purified and then directly sequenced in both directions by the company Genoscreen (Lille, France) on a SANGER technology platform using the primer pair BL18SPPF1/BL18SR2PP. For a significant percentage of fecal specimens, the obtained sequencing chromatograms exhibited double traces consistent with mixed infection, i.e., at least two *Blastocystis* sp. STs colonizing the same sample. STs present in these positive samples were not identified. The sequences obtained in the present study for single infections by *Blastocystis* sp. were deposited in GenBank under accession numbers PP357056–PP357090. All the sequences from samples presenting a single infection were aligned with each other using the BioEdit v7.0.1 package (http://www.mbio.ncsu.edu/BioEdit/bioedit.html (accessed on 1 January 2023)) to identify genetic variants within animal isolates and categorize them by so-called genotype. Subsequently, homologous sequences of human genotypes recently detected in the northern Egypt population [8] were extracted from GenBank and compared with genotypes reported herein from the cohorts of animals.

### 2.5. Phylogenetic Reconstruction and Subtyping

For subtyping of the animal isolates identified in the present study, an extensive dataset was created including 44 full-length SSU rDNA gene sequences extracted from GenBank and representatives of the different STs and subgroups of *Blastocystis* sp. [30,31]. To this reference framework were subsequently added 58 homologous sequences, each of which was representative of a genotype identified herein from camels, sheep, goats, horses, and/or rabbits. Multiple sequence alignment was performed using the program MAFFT v7.490, with the L-INS-i method employed for its robustness in aligning a panel of homologous sequences with one conserved domain and long gaps [51]. The maximum-likelihood (ML) phylogenetic tree was constructed with IQ-TREE [52] using the K2P+I+G4 substitution model with unequal transition/transversion rates and equal base frequency and supported by 1000 bootstrap replicates. Rooting of the tree was based on STs (ST15 and ST28) identified as the earliest emerging lineages within the *Blastocystis* genus in recent phylogenetic analyses [30,31,37]. ML-based phylogenetic placement of sequences was performed using EPA-ng [53], and the resulting data were processed and visualized using Gappa [54]. Additional enhancements and annotations for visualization were generated using the Interactive Tree Of Life (iTOL) platform for a more comprehensive depiction of phylogenetic relationships [55].

### 2.6. Statistical Analyses

Logistic regression models were established to examine the association between various categorical variables and to determine odds ratios (ORs) and 95% confidence intervals (CIs) considering *Blastocystis* sp. prevalence as the primary outcome. The *p*-value of 0.05 was selected as the limit for significance with 95% confidence intervals. All analyses were conducted using the “stats” and “oddsratio 2.0.1” packages in the R statistical computing program (https://www.r-project.org (accessed on 1 September 2023)).

## 3. Results

### 3.1. Analysis of the Animal Cohorts

We obtained 258 fecal samples from goats (breed Baladi) (103 males and 155 females) living in the Dakahlia (*n* = 111), Gharbia (*n* = 69), and Sharqia (*n* = 78) governorates. Animals of which 19 suffered from diarrhea were between 2 and 42 months old (mean age of 17.4 +/− 11.5 months) and categorized into four groups according to their age (up to 6 months, between 7 and 12 months, between 13 and 24 months, and more than 24 months). Regarding sheep (breed Saidi), 229 fecal specimens were collected from 96 males and 133 females in the Dakahlia (*n* = 88), Kafr El Sheik (*n* = 52), and Sharqia (*n* = 89) governorates. The ages of the animals (34 of them with diarrhea) ranged from 3 to 36 months (mean age of 14.8 +/− 9.4 months), with three age classes (up to 6 months, between 7 and 12 months, and over 12 months). The feces of 222 camels (95 males and 127 females including 12 animals diagnosed with diarrhea) were also collected in the Aswan (*n* = 76), Giza (*n* = 57), and Sharqia (*n* = 89) governorates. The average age of the camels was 3.6 +/− 1.5 years, and two age categories were selected (up to 3 years and over 3 years). Concerning horses (breed Baladi), fecal samples were obtained from 153 animals (77 males and 76 females) aged from 1 to 8 years (mean age of 3.7 +/− 1.9 years) and raised in the Dakahlia (*n* = 54), Gharbia (*n* = 42), and Sharqia (*n* = 57) governorates. Amongst these horses, eight of them presented diarrheic stools. Our specimen collection was completed by 227 fecal samples of rabbits from 3 to 24 months old (mean age of 9.7 +/− 7.3 months) obtained in the Dakahlia (*n* = 104), Sharqia (*n* = 68), and Kafr El Sheik (*n* = 55) governorates. The rabbit samples comprised the following breeds: New Zealand (*n* = 84), Flander (*n* = 45), Rex (*n* = 54), and Baladi (*n* = 44). Of these 227 rabbits, 24 were affected by diarrhea.

### 3.2. Occurrence of Blastocystis sp. in Targeted Animal Groups and Risk Factors of Infection

Out of a total of 1089 fecal samples collected from camels, horses, goats, sheep, and rabbits, the qPCR assay used herein revealed that 409 (37.6%) were positive for *Blastocystis* sp. (Table 1). However, significant differences in parasite frequency were observed between the different groups of animals examined. Indeed, the frequency of infection was particularly high in sheep (150/229, 65.5%), camels (138/222, 62.2%), and goats (93/258, 36.0%), while it was much lower in rabbits (23/227, 10.1%) and horses (5/153, 3.3%).

Data obtained from animal groups exhibiting the highest occurrences of the parasite (sheep, goats, and camels) were investigated through statistical analyses with the aim of estimating the influence of selected variables (sex, age, governorate, and stool consistency) on *Blastocystis* sp. infection (Table 2).

The sex was shown to have a significant effect on parasite colonization in sheep (prevalence of 71.4% in females versus 57.1% in males) where the risk of *Blastocystis* sp. infection was thus 1.5 times higher in the female group but not in goats (37.4% in females versus 34.0% in males) and camels (63.0% in females versus 61.0% in males). The collecting geographical area was also a variable found to have an impact on the risk of parasite infection in sheep since a significant higher prevalence was highlighted in the Kafr El Sheikh governorate (80.8%) compared with those reported in the Dakahlia (63.6%) and Sharqia (58.4%) governorates. Consequently, sheep from Kafr El Sheik had a 2.6 times higher risk of *Blastocystis* sp. infection than sheep from other governorates. Similarly, the number of goats colonized by the parasite was significantly lower in the Sharqia governorate (21.8%) and higher in the Dakalhia (45.0%) governorate since the risk of infection increased twofold in the last group. The frequency of *Blastocystis* sp. in camels was approximately twice lower in the Aswan governorate (35.5%) than in the Giza (70.2%) and Sharqia (79.8%) governorates, and thus, animals from Sharqia had a 4 times higher risk of *Blastocystis* sp. infection.

The age of the animals was not identified as a significant factor influencing the prevalence of *Blastocystis* sp. in either goat, sheep, or camels based on age categories considered. However, a higher frequency of the parasite was recorded in camels under 3 years of age compared with those over 3, in goats up to 24 months of age compared with older animals, and in sheep over 6 months of age compared with younger animals. Even though the number of animals suffering from diarrhea was limited (19/258, 34/229, and 12/222 in goats, sheep, and camels, respectively), the frequency of the parasite was reported to be higher in diarrheic goats (47.4% versus 35.1% for animals without diarrhea). Conversely, this prevalence was much lower in diarrheic camels compared with animals without digestive disorders (41.7% versus 63.3%) and in the same range between diarrheic sheep (64.7%) and animals without diarrhea (65.6%). However, these differences were not statistically significant.

### 3.3. Phylogenetic Analysis of Blastocystis sp. Isolates

**Figure 2:**
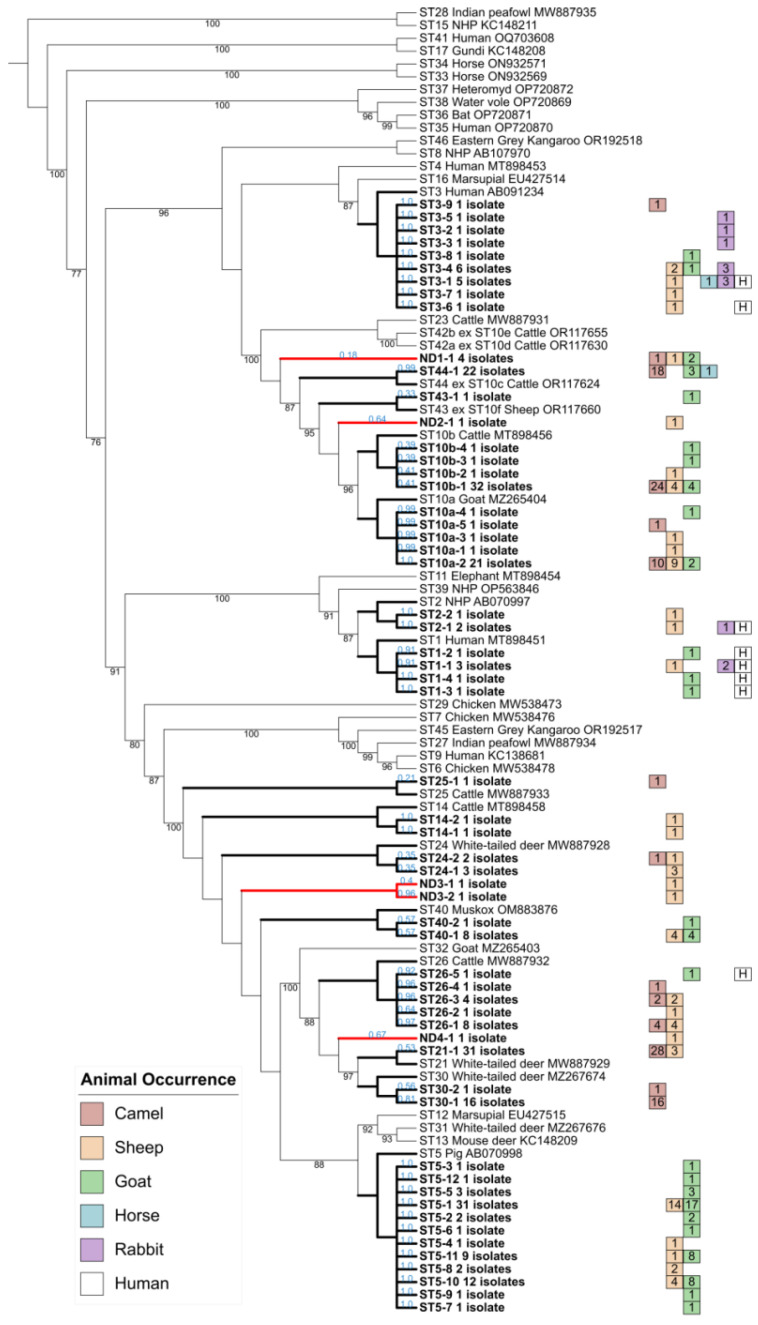
Maximum likelihood phylogenetic analysis of *Blastocystis* sp. isolates from animal groups based on SSU rDNA gene sequences. The phylogenetic tree is rooted using the reference sequences of the earliest diverging lineages ST15 and ST28 within the *Blastocystis* genus. Sequences from herbivores obtained in the current study (camel, sheep, goat, horse, and rabbit) are in bold, with the number of isolates from each animal type depicted by color-coded squares: red for camel, yellow for sheep, green for goat, blue for horse, and violet for rabbit. Isolates that are positioned within a specific ST are connected by black branches, while those whose ST designation is not confidently determined are connected by red branches (ND). Human-associated genotypes are indicated with white squares. Accession numbers for the reference sequences of known STs are provided. Bootstrap values are shown in black at the nodes of the tree, and values below 70% are not displayed. The likelihood of each sequence placement is indicated in blue next to the respective isolate.

The 409 qPCR products from positive samples were purified and directly sequenced. Among them, 146 (35.7%) were identified as mixed infections (at least two different STs colonizing the same animal) according to sequencing chromatogram analysis. The percentage of mixed infections varied widely according to animal group: 21% (29/138) in camels, 25.8% (24/93) in goats, 47.8% (11/23) in rabbits, and 52.7% (79/150) in sheep. It even reached 60% in horses but based on an extremely limited number of five positive samples. The remaining 263 chromatograms, derived from samples of the five animal groups studied, corresponded to single infections with *Blastocystis* sp. The corresponding sequences were aligned with each other, and a total of 58 genetic variants or genotypes were identified. These different genotypes were represented by a variable number of isolates ranging from 1 to 32. For subtyping of the corresponding animal isolates, these 58 genotypes were integrated into a broad phylogenetic analysis also including the 44 homologous reference sequences for each of the known STs and subgroups of *Blastocystis* sp. (Figure 2).

As highlighted in our phylogenetic tree, all the genotypes identified herein were successfully subtyped, except for five of them, grouped into four undetermined STs and designated ND1, found in sheep (one isolate), camels (one isolate), and goats (two isolates), and ND2, ND3, and ND4, each of which was only reported in one sheep sample. Of the remaining 53 genotypes, 38 were identified in only one of the five animal groups surveyed, 10 in two groups, and 5 in three groups. Furthermore, by comparing recently identified human genotypes in the population of northern Egypt with those of animals, we showed that eight genotypes were shared by animals and humans. More precisely, four human genotypes were common with goat, one with sheep, two with both sheep and rabbit, and one with sheep, horse, and rabbit.

### 3.4. Distribution of Blastocystis sp. STs in Animal Groups

On the basis of phylogenetic considerations, the distribution of *Blastocystis* sp. STs and subgroups was established for each of the animal groups (Table 3).

Regarding the sheep cohort, nine identified STs (ST1, ST2, ST3, ST5, ST14, ST21, ST24, ST26, and ST40) as well as the two subgroups ST10a and ST10b of *Blastocystis* sp. were detected. Among them, ST5 and ST10a were globally predominant even if all ST5 isolates were identified in the governorate of Dakahlia alone. In addition, the genetic diversity of isolates was markedly broader in the governorate of Sharqia (nine STs and subgroups) than in those described in the Dakahlia and Kafr El Sheikh (five STs and subgroups for each of them) governorates. In the case of the goat cohort, isolates were reported as belonging to ST1, ST3, ST5, ST26, ST40, ST43, ST44, and subgroups ST10a and ST10b. ST5 was largely predominant by representing more than 60% of the isolates subtyped in this animal group. Moreover, the range of STs and subgroups identified in the Dakahlia governorate (nine in total) was much wider than those evidenced in the Gharbia (two) and Sharqia (only one) governorates. In camels, a total of eight STs (ST3, ST21, ST24–ST26, ST30, and ST44) and two subgroups (ST10a and ST10b) were identified with varying frequencies and with a large majority of isolates belonging to ST10a and b, ST21, ST30, and ST44. This variation in distribution and frequency of the STs was reported between governorates since seven STs and subgroups were identified in the Giza governorate, six in the Sharqia governorate, and only four in the Aswan governorate. Additionally, all ST21 isolates were reported in the Sharqia governorate, whereas ST3, ST24, and ST25 were only found in the Giza governorate. The 12 isolates subtyped in rabbits originated from ST1, ST2, and ST3, with ST3 predominant in each of the three investigated governorates (Sharqia, Dakahlia, and Kafr El Sheikh), whereas the only two isolates subtyped in horses belonged to ST3 and ST44.

### 3.5. Assessment of Intra-ST Diversity of Blastocystis sp. Isolates

**Figure 3:**
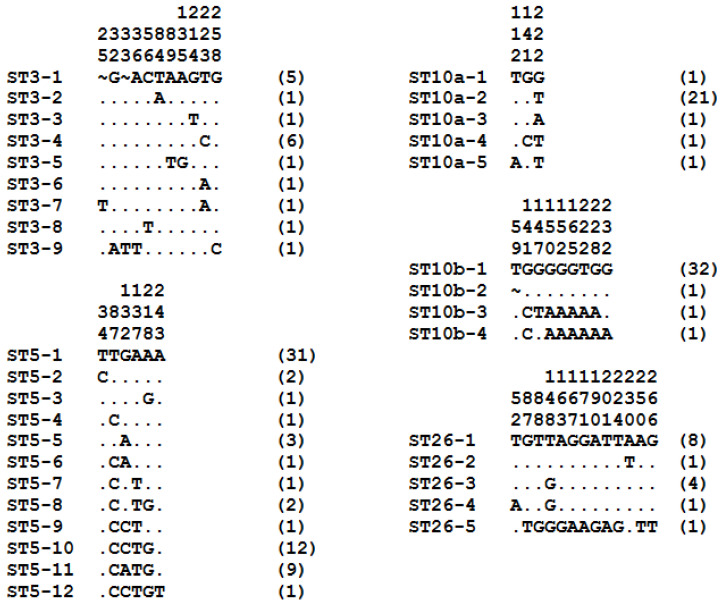
SSU rDNA gene sequence polymorphism between genotypes of some of the most frequently STs and subgroups identified in the present study. The variable positions reported between sequences of isolates belonging to ST3, ST5, and ST26 as well as subgroups ST10a and ST10b are indicated above the alignment (vertical numbering) with respect to the arbitrarily selected reference sequences called genotype STX-1. Nucleotides identical to those of the reference sequences are represented by dashes, and gaps are represented by asterisks. The number of isolates for each genotype is indicated in parentheses on the right of the alignments.

The subtyping data were supplemented by an assessment of intra-ST diversity through the analysis of sequence polymorphism of isolates belonging to the major STs and subgroups including more than 10 isolates identified in the present survey (ST3, ST5, ST10a, ST10b, ST21, ST26, ST30, and ST44) (Figure 3).

The average ratio of the number of isolates per genotype that is inversely proportional to intra-ST variability was calculated for each of these STs and subgroups. The lowest intra-ST diversity was evidenced for ST21 with an average ratio of 31 isolates/genotype (31/1) and ST44 (average ratio of 22 isolates/genotype, 22/1), followed by ST10b (8.7, 35/4), ST30 (8.5, 17/2), ST5 (5.4, 65/12), ST10a (5, 25/5), ST26 (3, 15/5), and ST3 (2, 18/9).

## 4. Discussion

To the best of our knowledge, the present study represented the largest molecular-based survey ever carried out on the epidemiology of *Blastocystis* sp. in animals in Egypt and more globally in North Africa in terms of the number of fecal samples screened using molecular tools and subtyped isolates. The study was conducted in six Egyptian governorates, and a total of 1089 stool specimens collected from camels, rabbits, goats, sheep, and horses were analyzed for the presence of the protozoa, leading to the subtyping of 263 isolates. Interestingly, this study succeeded those carried out in northern Egypt using the same molecular marker in additional animal cohorts (poultry, cattle, and pets) [48] as well as in the human population [8], thus expanding our knowledge on the ST distribution and circulation of *Blastocystis* sp. in this geographical area. In addition, given that the groups of animals investigated in the present study interacted with the local population, the obtained molecular data allowed us to assess the zoonotic potential of this protozoan.

Overall, of the 1089 stool samples tested for the five animal groups, *Blastocystis* sp. was identified in 409 of them by qPCR, i.e., a prevalence of 37.6%, thus highlighting a very active circulation of the parasite in Egypt. However, the frequency of infection was extremely variable from one group of animals to another, ranging from 3.3% in horses to 65.5% in sheep. By analyzing our molecular data group by group, the highest frequency of the parasite was thus observed within the sheep cohort. A comparable prevalence has been documented in surveys examining a small number of fecal samples from this animal group and conducted in China (53.8%) [56], United Arab Emirates (63.6%) [57], and Portugal (60.9%) [58]. This frequency was therefore well above the average infection rate in sheep, estimated at around 25% worldwide from a systematic review and meta-analysis including over 3000 animals tested in 10 countries [59], thus emphasizing a frequent colonization of small ruminants in Egypt. Prevalence in sheep varied considerably among the Egyptian governorates, ranging from 58.4% to 80.8%. Significant differences in frequency related to the collecting geographical area within the same country have also been observed in sheep cohorts, particularly in China, as recently reviewed [60]. These regional disparities may be related to a variety of factors including, among others, the season of collection [61], the sanitary rules applied on each livestock farms, and different patterns of sheep feeding and rearing facilitating parasite transmission, as illustrated by the comparison of *Blastocystis* sp. prevalence on intensive farms and in grazing models in China (23.8% versus 4.1%) [60]. With respect to age, no significant difference in prevalence was herein reported in sheep by comparing the three age cohorts considered, in agreement with other surveys carried out in China on this same group of animals [60,62,63]. On the other hand, the sex was shown to have a significant effect on parasite colonization in sheep (prevalence of 71.4% in females versus 57.1% in males). To explain the higher frequency of gastrointestinal parasites in female livestock animals among small ruminants, it has been suggested that stress during pregnancy and lactation could predispose them to infectious diseases by altering their immunity [64].

On the basis of recent reviews and supplementary epidemiological studies [12,31,39,58,59,63], no fewer than 20 STs have been currently identified in sheep worldwide including ST1–ST7, ST10, ST12, ST14, ST15, ST21, ST23–ST26, ST30, and ST42–ST44. The phylogenetic analysis performed in the present study allowed us to confirm the wide genetic diversity of isolates among single infections in sheep with the identification of 10 STs (ST1–ST3, ST5, ST10 (both subgroups a and b), ST14, ST21, ST24, ST26, and ST40), 9 of them already detected in previous surveys. Consequently, our study represented the first description of ST40 in sheep, providing an update to the host range of this ST so far only detected in muskoxen [65]. Together, ST5 and ST10 (both a and b) isolates accounted for around 60% of the subtyped isolates in this animal group in Egypt. Both STs were also largely predominant in surveys conducted in China [60,63] and Iran [66]. In addition, ST10 was also the most frequently ST found in another Chinese study [56], in Greenland [65], and in Portugal [58] as in a first survey performed in the United Arab Emirates [57]. ST10 was also the only ST reported in sheep from Italy [67], as well as in a subsequent survey carried out in United Arab Emirates [68]. ST14 represented another frequent ST identified in sheep worldwide as evidenced in China [61] and Portugal [58], while only two Egyptian isolates (3%) were shown to belong to this ST in the present study. In accordance with all these data, sheep would therefore be primarily colonized by ST5, ST10, and ST14 as shown in an Iranian survey [69]. With the exception of ST1 to ST4 and ST6 and ST7, the other STs likely adapted to ruminants appeared to be less frequent findings with prevalence mainly depending on geographical area even if, in Portugal, ST24, ST43, and ST44 were among the most common STs identified [58]. The diversity of STs reported and their prevalence also proved highly variable from one governorate to another, as clearly demonstrated particularly by the identification of ST5 in the sole governorate of Dakahlia. Similar observations regarding varying ST distributions were reported in multi-region studies conducted in China [60,63] presumably related to environmental differences between collecting areas.

In the group of goats under investigation in our study, 36.0% of the animals were found to be colonized by the parasite, an occurrence close to the average infection rate (28.3%) calculated from around 2300 goats examined in 13 studies conducted worldwide [59] or, more recently, reported from a population of over 200 black goats in China (33.6%) [70]. No effect of sex or age on *Blastocystis* sp. infection was apparent in goats. In contrast, a variation in parasite frequency was observed according to governorate (21.8% to 45.0%), which may be essentially due to rearing and sanitary conditions [60]. As for sheep, the existing molecular data collected in many parts of the world indicated that goats may carry numerous potential STs, including ST1, ST3–ST8, ST10, ST12–ST14, ST21, ST23–ST26, ST30, ST32, ST43, and ST44 [12,14,31,39,59]. Seven of these established STs were also detected among the goats examined herein (ST1, ST3, ST5, ST10 (a and b), ST26, ST43, and ST44). Interestingly, an additional ST was identified in our cohort of animals that has not previously been reported to the best of our knowledge in goats, namely, ST40, which was found to be common in muskoxen in northeast Greenland [65]. As described above, ST40 was also found in sheep of Egypt, thus broadening the range of small ruminant hosts potentially colonized by this ST. Regarding the frequency of STs in our group of goats, ST5 was largely predominant in each governorate and globally included nearly 65% of subtyped isolates. It was followed by ST10 (a and b) only identified in the Dakahlia governorate, while the other STs reported were scarcely represented in terms of number of isolates. ST5 was also the most common ST identified in goats as in recent surveys conducted in Malaysia [14] and Italy [67]. ST10 was the second most frequent ST found in our goat cohort also featuring the absence of ST14. However, ST10 was reported as the commonest ST in numerous surveys followed by ST14 [59,60,70] or ST24 [58]. ST10 and ST14 may even be the only two STs detected in the goats in certain studies [56].

In the current study, camels emerged as the second most infected animal group, exhibiting a prevalence exceeding 60%. This prevalence notably surpassed prior findings in the Ismailia governorate of northeastern Egypt, where a prevalence of 25.0% was reported from a small sample group of 20 animals [45]. For the other two epidemiological surveys available in the literature involving a significant number of camel samples, prevalence rates of 24.0% (47/196) in Libya [38] and 21.8% (139/638) in China [71] were observed. In our camel cohort, neither sex nor age represented significant risk factors for parasite infection. In contrast, notable variation in *Blastocystis* sp. frequency was reported among different collection areas, with rates ranging from 35.5% in the Aswan governorate to a striking 79.8% in the Sharqia governorate. Similar variations among collection sites have been underscored in a Chinese survey, where *Blastocystis* sp. prevalence ranged from 0% to 70.6% [71], likely influenced by geographical factors and differing animal management practices across regions. To date, only four surveys worldwide have provided subtyping data from camel isolates [38,45,71,72], revealing the identification of 14 different STs including ST1, ST3–ST7, ST10, ST14, ST15, ST21, ST24–ST26, and ST30. From this limited body of published studies, ST10 exhibited a slight predominance, closely followed in frequency by ST30, ST14, ST5, ST24, and ST25, while remaining STs were sporadically identified. In our survey, we found that camels were colonized by a variety of STs including ST3, ST10 (a and b), ST21, ST24, ST25, ST26, ST30, and notably, ST44, which is being reported for the first time in camels. In agreement with the aforementioned synthesis, ST10 predominated in our survey, with ST21, ST44, and ST30 trailing behind in frequency. Nevertheless, the prevalence of certain STs was markedly influenced by the collection area. Notably, all ST21 isolates were confined to the Sharqia governorate.

After examining the three ruminant groups, the remaining two groups screened consisted of non-ruminant herbivores, specifically horses and rabbits. To our knowledge, around a dozen surveys have been conducted to detect *Blastocystis* sp. in horses, with the majority involving no more than thirty animals. In several of these studies, the parasite was not identified, as in Libya and the United Kingdom [38], China [73], Turkey [74], Iran [69], and Portugal [58], or was present at a very low prevalence, as in Thailand (3.5%) [75]. This corroborated our findings, as only 5 out of the 153 tested horses were found to be positive for *Blastocystis* sp. (3.3%), strongly indicating a restricted circulation of the parasite within this group of animals worldwide. Strikingly, a recent survey conducted in Colombia, involving nearly 200 horses, revealed a prevalence exceeding 40% [76], confirming findings from a prior study performed in the same country, which reported a frequency of over 50% in a group of around a dozen horses [77]. The authors suggest that such variations in infection rate could be attributed to various factors, including host-related factors such as breed, as well as the diagnostic method employed to detect *Blastocystis* sp.—although in our survey as in others, PCR was used for this purpose. Additionally, factors like water and feed sources should also be considered. Given the low prevalence of the parasite within our equine cohort, only two isolates were subtyped, revealing they belonged to ST3 and ST44. Previous studies have identified 13 different STs in horses including ST1–ST6, ST10, ST14, ST24–ST26, ST33, and ST34 [56,72,76,77], primarily through research conducted in Colombia, and with a significant predominance of ST10. Our investigation thus added ST44 to the list of STs that could potentially colonize horses. Undoubtedly, prevalence and ST data remain too scarce in horses, and further research across equine populations is imperative to assess the situation in various geographical areas and enrich our understanding of the molecular epidemiology of *Blastocystis* sp. in this animal group.

The last group of animals examined was rabbits, with a reported overall prevalence of 10.1% in Egypt. Specifically, the frequency of *Blastocystis* sp. was similar between rabbit breeds (New Zealand, 8/84, 9.5%; Flander, 5/45, 11.1%; Rex, 4/54, 7.4%; Baladi, 6/44, 13.6%). By focusing solely on surveys restricted to China and Spain that had previously analyzed a substantial number of rabbit samples, the reported prevalence was comparable to our study, ranging between 1.0% and 15.0% [73,78,79,80,81]. Simultaneously, a study conducted in northern Egypt (governorate of Ismailia) on about ten rabbits did not reveal the presence of the parasite [45] as among a group of a hundred animals examined in Australia [30]. As a result, all these data suggested a limited circulation of *Blastocystis* sp. within the Leporidae. A total of seven STs were previously reported in rabbits with varying frequencies, including ST1–ST4, ST7, ST14, and ST17 [57,73,78,80,81]. Three of these STs (ST1, ST2, and ST3) were identified in the current study among rabbits, with a higher prevalence of ST3 isolates.

Based on all the molecular data collected in the present study, hypotheses could be proposed regarding the circulation of *Blastocystis* sp. among these five animal groups in Egypt, even though certain isolates could not be subtyped. Indeed, four genotypes, designated ND1 to ND4, colonizing goats, sheep, and/or camels, displayed an uncertain position in our phylogenetic analysis. Two explanations could account for this uncertainty. The first involved the lack of resolution of the SSU rDNA gene domain, used as a marker in the qPCR assay, due to its reduced length (around 300 bp) in comparison with the full-length sequence of the gene. The second possibility was that these genotypes might represent new STs. To validate either of these assumptions, producing the complete sequence of the SSU rDNA gene of representatives of these four genotypes using the Nanopore sequencing strategy [76,82] would be mandatory in further investigations.

In Egypt, ruminants, encompassing sheep, goats, and camels, were thus consistently colonized by *Blastocystis* sp., with the identification of an extensive range of STs circulating in this animal population. Most of these STs are likely adapted to these hosts, such as the predominant ST10 and ST14 [12], together with ST21, ST23–ST26, ST30, ST40, ST43, and ST44 originally identified in ruminants [58,65,77,82]. Indeed, phylogenetic analyses based on full-length SSU rDNA gene sequences documented a common ancestry for ST14 and ST21, ST24–ST26, ST30, and ST40 on one side and ST10 and ST23, ST43, and ST44 on the other [31,82], supporting their likely host preference to ruminants. Furthermore, our survey revealed the occurrence of various genotypes, belonging to ST10, ST21, ST24, ST26, and ST40, shared by two or three of the tested ruminant groups, evidencing the widespread circulation of the same isolates and possibly cross-transmission events between ruminants exhibiting the same physiology of the digestive tract and a common habitat. As evidenced through the analysis of the intra-ST diversity of isolates, the only genetic variant identified for ST21 was colonizing sheep and camels, and the genetic variants ST10a-2 and ST10b-1 representing together approximately 90% of the ST10 isolates were both identified in sheep, goats, and camels. Although several STs were thus common to all three ruminant groups herein, albeit with varying frequencies as ST10, some STs could also exhibit a host preference within ruminants. Indeed, ST5 and ST40 were only found in small ruminants, while ST21, ST30, and ST44 were solely or largely predominantly identified in camels. It was also noteworthy to mention that ST5, which was reported with a high frequency in omnivores and specially Suidae [12,44], can also significantly infect small ruminants (sheep and goats) in Egypt and beyond, as previously highlighted [60,63].

The potential impact of animal diet on the rate of *Blastocystis* sp. infection and host suitability was herein strongly suggested. Indeed, in horses and rabbits, which are herbivores but not ruminants (monogastric versus polygastric), the frequency of the parasite decreased to 3.3% and 10.1%, respectively, and therefore was significantly lower than that observed in the ruminant groups analyzed in our study from the same geographical area and thus with potential exposure to similar environmental sources. In this context, it has also been demonstrated that *Blastocystis* sp. was more prevalent among omnivores and herbivores compared with carnivores, where colonization was a rare occurrence overall [12,13,15,39]. This was demonstrated, for instance, in pets in northern Egypt [48], strongly suggesting that dietary preference and diversity of digestive systems could potentially influence parasite colonization rates and ST distribution/preference. Additional factors including genetics, physiological, and/or immunological barriers or interaction with the gut microbiome of the host might account for the host specificity/preference of *Blastocystis* sp., which remains still poorly understood [26,83].

The low frequency of the parasite reported in horses and rabbits prevented the identification of truly predominant STs in these two animal groups. Based on single infections subtyping data, only two STs, ST3 and ST44, were identified herein in horses and only three, ST1–ST3, in rabbits. As stated above, ST44 has been primarily identified in camels in our survey, and its sporadic identification (only one isolate) in horses and for the first time in equines was likely to be attributed to accidental contamination through a contact with ruminant feces. Moreover, almost all epidemiological studies have unanimously concluded that ST1–ST4 represented the main STs of human infection reported worldwide [33,39]. This was also the case in northern Egypt, where a recent study showed a prevalence of *Blastocystis* sp. exceeding 70%, with a considerable predominance of ST1–ST3 (98.6% of subtyped isolates) [8]. Consequently, although detected in other animal hosts [12,39], these STs might be considered to be of “human origin” due to their high prevalence and circulation in the population [1]. Thus, the presence of one isolate of ST3 in horses and 12 isolates belonging to ST1, ST2, or ST3 in rabbits could likely be explained by the occurrence of reverse zoonosis transmission from human handlers to animals as suggested in previous studies [12]. A similar explanation could be proposed for the few ST1–ST3 isolates identified herein in ruminants (eight isolates in sheep, five in goats, and one in camels). In this sense, our phylogenetic analysis revealed that two of the nine ST3 genotypes, one of the two ST2 genotypes, and the four ST1 genotypes identified in goats, sheep, rabbits, and horses in the present survey were shared with the human population living in the same geographical region of Egypt and thus potentially in contact with these animals [8]. Interestingly, ST2 was reported in waste water from various sources in the Dakahlia governorate that is discharged in the Nile River after minimal treatments [84]. Given its utilization in the irrigation of agricultural lands in some Egyptian rural areas, as well as its potential use for animal feed, this water source may serve as a reservoir for *Blastocystis* sp. contamination among herbivores and other animal populations.

The role of these five groups of herbivore livestock animals in the transmission of *Blastocystis* sp. to humans was assessed through the identification of the isolates as potentially zoonotic. Evidence of a zoonotic event was first gained from the finding of the same ST26 genotype in a goat and an individual also living in the governorate of Dakahlia [8], although it was not known whether this participant stated direct contact with ruminants. However, ST26 could be transmitted indirectly via water consumption since it has recently been detected in rain collection vessels, a source of drinking water in a rural Thai community [35]. To our knowledge, this corresponded to the first report of ST26 in the human population, thus extending the host range of this specific ST. By taking into account our current discovery of ST26 in the Egyptian population together with STs previously classified as zoonotic because they were recorded in both humans and animals (ST1–ST10, ST12, ST14, ST16, ST23, and ST35) [6,8,29,33,34,35,36], an impressive rate of 66.3% (169/255) of animal isolates subtyped in the present study was recorded as potentially transmissible to humans. More precisely, this proportion amounted to 83.3% in sheep (55/66), 86.6% in goats (58/67), and 39.8% (43/108) in camels. Based on a limited number of isolates from other herbivores, this ratio reached 50% (1/2) in horses and 100% (12/12) in rabbits. These findings hold significant implications for public health, shedding light on the pivotal role that these animal groups may play in the transmission of zoonotic STs to humans. However, to overcome the limitations of the present survey only focused on animals, the global public health outcome and transmission dynamics of the parasite have to be deciphered at the human–animal–environment interface in the context of a One Health perspective [35]. Further investigations are thus currently in progress in Egypt aiming to detect simultaneous *Blastocystis* sp. in animals (livestock), humans (breeders and any other population in contact with these animals), and environmental (water and soil) samples as in food matrices in the same restricted geographical area.

Finally, the overall proportion of animals exhibiting diarrhea was limited in the present study. Interestingly, a higher prevalence of the parasite was highlighted among diarrheic goats compared with goats without digestive disorders, but this was not the case for sheep and even less so for camels, where *Blastocystis* sp. was less frequent in diarrheic animals than in healthy ones. Therefore, no clear association could be established between the presence of the parasite and the development of gastrointestinal symptoms based on our limited and conflicting data across animal groups. The recent literature confirms that the majority of *Blastocystis* sp. infections in animal populations show no correlation with clinical signs of disease [12]. However, this topic remains largely unexplored in animals, and further studies have to be conducted considering simultaneously the genetic diversity of *Blastocystis* sp. isolates and the immune status and composition of the intestinal microbiota of the animals, along with the identification of other co-existing pathogens that may potentially induce similar digestive disorders. Moreover, *Blastocystis* sp. could also exert an indirect effect likely by inducing modifications in the composition of the host gut microbiota, thereby facilitating the colonization of pathogenic bacteria, as demonstrated in poultry, for which a significant positive correlation was reported between the occurrence of *Campylobacter* spp. and the protozoan [85].

## 5. Conclusions

This large-scale survey conducted in Egypt provided new insights into the prevalence, genetic diversity, host specificity, and zoonotic potential of *Blastocystis* sp. in herbivores closely interacting with the human population. This protozoan was identified as a common finding in small ruminants including sheep, goats, and camels highlighting active circulation within these animal groups, while horses and rabbits showed lower rates of colonization by the parasite. Sheep, goats, and camels harbored a great diversity of STs, most of them likely adapted to ruminants and thus shared by all three animal groups. Given the notably high frequencies of *Blastocystis* sp., particularly among ruminants, and the substantial proportion of animal isolates subtyped herein as potentially zoonotic, preventive measures have to be implemented in Egypt with the aim to reduce parasite circulation and, consequently, the risk of infection in both humans and animals.

## Figures and Tables

**Table 1:** Prevalence of *Blastocystis* sp. in animal groups sampled in different Egyptian governorates.

Host	Governorate	Samples (*n*)	Positive Samples (*n*)	Prevalence (%)
Sheep	Sharqia	89	52	58.4
	Dakahlia	88	56	63.6
	Kafr El Sheikh	52	42	80.8
**Total**		**229**	**150**	**65.5**
Goat	Sharqia	78	17	21.8
	Dakahlia	111	50	45.0
	Gharbia	69	26	37.7
**Total**		**258**	**93**	**36.0**
Camel	Sharqia	89	71	79.8
	Aswan	76	27	35.5
	Giza	57	40	70.2
**Total**		**222**	**138**	**62.2**
Horse	Sharqia	57	0	0
	Dakahlia	54	1	1.8
	Gharbia	42	4	9.5
**Total**		**153**	**5**	**3.3**
Rabbit	Sharqia	68	10	14.7
	Dakahlia	104	9	8.6
	Kafr El Sheikh	55	4	7.3
**Total**		**227**	**23**	**10.1**
**Grand total**		**1089**	**409**	**37.6**

**Table 2:** Variables associated with frequency of *Blastocystis* sp. in sheep, goats, and camels from Egypt.

Animal Group/Variable	No. Tested	No. Positive (Prevalence)	OR (95% CI)	*p*-Value
**Sheep**	**229**	**150 (65.5%)**		
Sex				
Male	96	55 (57.3%)	OR: 0.537, CI: 0.308–0.931	0.027 *
Female	133	95 (71.4%)	OR: 1.463, CI: 1.069–1.692	0.027 *
Governorate				
Dakahlia	88	56 (63.6%)	OR: 0.875, CI: 0.502–1.534	0.639
Kafr El Sheik	52	42 (80.8%)	OR: 2.683, CI: 1.308–5.978	0.011 *
Sharqia	89	52 (58.4%)	OR: 0.602, CI: 0.345–1.050	0.074
Age (months)				
<6	52	31 (59.6%)	OR: 0.719, CI: 0.382–1.372	0.311
7–12	84	57 (67.9%)	OR: 1.180, CI: 0.671–2.103	0.568
>12	93	62 (66.7%)	OR: 1.091, CI: 0.627–1.913	0.759
Stool consistency				
Solid	195	128 (65.6%)	OR: 1.042, CI: 0.473–2.203	0.916
Diarrheic	34	22 (64.7%)	OR: 0.960, CI: 0.454–2.113	0.916
**Goat**	**258**	**93 (36.0%)**		
Sex				
Male	103	35 (34.0%)	OR: 0.861, CI: 0.508–1.446	0.573
Female	155	58 (37.4%)	OR: 1.139, CI: 0.554–1.492	0.573
Governorate				
Dakahlia	111	50 (45.0%)	OR: 1.982, CI: 1.186–3.333	0.009 *
Gharbia	69	26 (37.7%)	OR: 1.101, CI: 0.617–1.940	0.741
Sharqia	78	17 (21.8%)	OR: 0.381, CI: 0.202–0.692	0.002 *
Age (months)				
>6	59	22 (37.3%)	OR: 1.072, CI: 0.581–1.946	0.821
7–12	78	27 (34.6%)	OR: 0.914, CI: 0.520–1.587	0.753
13–24	62	26 (41.9%)	OR: 1.391, CI: 0.771–2.489	0.269
>24	59	18 (30.5%)	OR: 1.391, CI: 0.771–2.489	0.269
Stool consistency				
Solid	239	84 (35.1%)	OR: 0.602, CI: 0.234–1.571	0.290
Diarrheic	19	9 (47.4%)	OR: 1.661, CI: 0.637–4.277	0.290
**Camel**	**222**	**138 (62.2%)**		
Sex				
Male	95	58 (61.0%)	OR: 0.921, CI: 0.533–1.596	0.768
Female	127	80 (63.0%)	OR: 1.079, CI: 0.404–1.467	0.768
Governorate				
Aswan	76	27 (35.5%)	OR: 0.174, CI: 0.094–0.315	1.37 × 10^−8^ *
Giza	57	40 (70.2%)	OR: 1.609, CI: 0.853–3.132	0.1498
Sharqia	89	71 (79.8%)	OR: 3.886, CI: 2.127–7.37	1.72 × 10^−5^ *
Age (years)				
<3	114	73 (64.0%)	OR: 1.178, CI: 0.684–2.031	0.554
>3	108	65 (60.2%)	OR: 0.849, CI: 0.492–1.461	0.555
Stool consistency				
Solid	210	133 (63.3%)	OR: 2.418, CI: 0.747–8.417	0.143
Diarrheic	12	5 (41.7%)	OR: 0.414, CI: 0.119–1.339	0.143

* Statistically significant.

**Table 3:** Distribution of STs and subgroups of *Blastocystis* sp. isolates sampled in the present survey per animal group and governorate.

Host	Governorate	*Blastocystis* sp. STs and Subgroups																			
		1	2	3	5	10a	10b	14	21	24	25	26	30	40	43	44	ND1	ND2	ND3	ND4	MI
**Sheep**	Sharqia	1	2	5	0	6	4	1	1	1	0	1	0	0	0	0	0	0	1	0	29
	Dakahlia	0	0	0	22	2	1	0	2	0	0	5	0	0	0	0	0	0	0	0	24
	Kafr El Sheikh	0	0	0	0	3	0	1	0	3	0	1	0	4	0	0	1	1	1	1	26
**Total**		**1**	**2**	**5**	**22**	**11**	**5**	**2**	**3**	**4**	**0**	**7**	**0**	**4**	**0**	**0**	**1**	**1**	**2**	**1**	**79**
**Goat**	Sharqia	0	0	0	15	0	0	0	0	0	0	0	0	0	0	0	0	0	0	0	2
	Dakahlia	3	0	1	13	3	6	0	0	0	0	1	0	5	1	3	2	0	0	0	12
	Gharbia	0	0	1	15	0	0	0	0	0	0	0	0	0	0	0	0	0	0	0	10
**Total**		**3**	**0**	**2**	**43**	**3**	**6**	**0**	**0**	**0**	**0**	**1**	**0**	**5**	**1**	**3**	**2**	**0**	**0**	**0**	**24**
**Camel**	Sharqia	0	0	0	0	1	10	0	28	0	0	3	6	0	0	12	0	0	0	0	11
	Aswan	0	0	0	0	2	11	0	0	0	0	1	11	0	0	0	0	0	0	0	2
	Giza	0	0	1	0	8	3	0	0	1	1	3	0	0	0	6	1	0	0	0	16
**Total**		**0**	**0**	**1**	**0**	**11**	**24**	**0**	**28**	**1**	**1**	**7**	**17**	**0**	**0**	**18**	**1**	**0**	**0**	**0**	**29**
**Horse**	Sharqia	0	0	0	0	0	0	0	0	0	0	0	0	0	0	0	0	0	0	0	0
	Dakahlia	0	0	0	0	0	0	0	0	0	0	0	0	0	0	1	0	0	0	0	0
	Gharbia	0	0	1	0	0	0	0	0	0	0	0	0	0	0	0	0	0	0	0	3
**Total**		**0**	**0**	**1**	**0**	**0**	**0**	**0**	**0**	**0**	**0**	**0**	**0**	**0**	**0**	**1**	**0**	**0**	**0**	**0**	**3**
**Rabbit**	Sharqia	1	1	4	0	0	0	0	0	0	0	0	0	0	0	0	0	0	0	0	4
	Dakahlia	0	0	2	0	0	0	0	0	0	0	0	0	0	0	0	0	0	0	0	7
	Kafr El Sheikh	1	0	3	0	0	0	0	0	0	0	0	0	0	0	0	0	0	0	0	0
**Total**		**2**	**1**	**9**	**0**	**0**	**0**	**0**	**0**	**0**	**0**	**0**	**0**	**0**	**0**	**0**	**0**	**0**	**0**	**0**	**11**
**Grand total**		**6**	**3**	**18**	**65**	**25**	**35**	**2**	**31**	**5**	**1**	**15**	**17**	**9**	**1**	**22**	**4**	**1**	**2**	**1**	**146**

MI, mixed infection.

## Data Availability

All relevant data are within the paper.
